# Polyphenol-Rich Beverages Exert Beneficial but Variable Effects on Oxidative, and Inflammatory Markers in Metabolic Syndrome and Related Conditions: Evidence of Human Trials

**DOI:** 10.3390/foods14244341

**Published:** 2025-12-17

**Authors:** Nevena Vidovic, Vuk Stevanovic, Milica Zekovic, Marija Takic

**Affiliations:** 1 Group for Nutrition and Metabolism, Centre of Research Excellence in Nutrition and Metabolism, National Institute of Republic of Serbia, Institute for Medical Research, University of Belgrade, 11000 Belgrade, Serbia; milica.zekovic@imi.bg.ac.rs (M.Z.); marija.takic@imi.bg.ac.rs (M.T.); 2 Group for Nutrition and Metabolism, National Institute of Republic of Serbia, Institute for Medical Research, University of Belgrade, 11000 Belgrade, Serbia; vuk.stevanovic@imi.bg.ac.rs

**Keywords:** polyphenols, metabolic syndrome, oxidative stress, inflammation

## Abstract

Metabolic syndrome (MetS), one of the major global health concerns, represents a cluster of cardiometabolic risk factors along with chronic low-grade inflammation and oxidative stress as essential features. Lifestyle changes, including the health quality of the foods, are recommended as the initial interventions for the management and eventual reversal of metabolic syndrome. Considering the heterogeneity of the studies in evaluating the health benefits of polyphenol-rich foods, there is a lack of quantitative and even qualitative analysis of their potential impact on this pathophysiological condition. This review aimed to provide a coherent, clinically oriented appraisal of the potential role of polyphenol-rich juices and beverages in the management of metabolic syndrome and related cardio-metabolic conditions. Twenty-three human intervention studies, encompassing randomized controlled, crossover, and parallel-group designs, as well as non-randomized or uncontrolled intervention studies that prospectively evaluated a defined beverage. The collective evidence indicates polyphenols could improve anthropometric parameters and blood lipid levels, while data on insulin and blood pressure seemed inconsistent and limited. Regarding the antioxidant effects, most beverages beneficially affected lipid peroxidation and total antioxidant activity. Findings across the studies portray polyphenol-rich juices and beverages as consistent, though not uniformly potent, modulators of low-grade inflammation in cardiometabolic contexts. To draw any firm conclusions, future trials are recommended. These should adopt consistent polyphenol quantification and dosage applied, standardize analyzed parameters including inflammatory and oxidative stress panels, stratify participants by baseline status and medication use, and extend follow-up to evaluate reliability and clinical significance.

## 1. Introduction

Metabolic syndrome (MetS), also known as “Syndrome X” and “Insulin Resistance Syndrome”, represents one of the major global public health concerns, as its prevalence keeps increasing [1]. According to mostly used criteria, defined by the National Cholesterol Education Program Adult Treatment Panel III, MetS diagnosis includes the presence of at least three among five risk factors: elevated waist circumference, elevated triacylglycerol (≥1.7 mmol/L), reduced high-density lipoprotein (HDL) cholesterol (<1.03 mmol/L for men and <1.29 mmol/L for women), elevated systolic and diastolic blood pressure (SBP/DBP > 130/85 mmHg), and elevated fasting glucose (≥5.6 mmol/L) [2]. Although the exact mechanisms underlying MetS pathophysiology are still not fully elucidated, it is well established that the condition is accompanied by increased oxidative stress and inflammation. Furthermore, most MetS subjects are obese, sedentary, and have insulin resistance at some level [1]. Increased adiposity, closely related to metabolic syndrome, promotes inflammation and oxidative stress, both of which contribute to the development of MetS components, including insulin resistance, dyslipidemia, and hypertension [3].

Subjects with metabolic syndrome often have lower plasma antioxidant activity, along with higher indicators of oxidative damage, compared to healthy individuals. This leads to the development of oxidative stress and contributes to decreased insulin sensitivity, a key feature of MetS [4]. As previously reported, the presence of MetS might aggravate oxidative stress in obese subjects and increase lipid peroxidation, i.e., levels of thiobarbituric acid reactive substances (TBARS) and hydroperoxides [5]. Compared to the age and sex matched controls, subjects with MetS might have decreased antioxidant defense with altered activities of antioxidant enzymes (including superoxide dismutase, glutathione reductase, glutathione peroxidase, and catalase) [6].

Besides oxidative stress, MetS has been linked to increased inflammatory burden. Metabolic syndrome is often accompanied by abdominal obesity and accumulation of visceral fat, which contribute to the production of both reactive oxygen species and proinflammatory molecules, such as TNF-alpha [7]. Accordingly, patients with MetS have been characterized with higher levels of plasma CRP, compared to control subjects [5].

Lifestyle modifications, including a healthy diet, exercise, and weight regulation, are advised as the first-line interventions for managing and potentially reversing MetS [8]. Polyphenols represent a large family of secondary metabolites with diverse chemical structures. There are more than 8 thousand structural variants of polyphenols, but all are characterized by the existence of one or more aromatic rings and one or more hydroxyl functional groups. Their structure varies from basic molecules to highly polymerized ones (like condensed tannins) [9]. The broadest group of polyphenols is flavonoids, consisting of two aromatic rings, linked by three carbon atoms, forming an oxygen-containing heterocyclic ring. Flavonoids can further be divided into six subclasses as a function of the type of heterocycle involved. Polyphenols are mainly detected in fruits, vegetables, and beverages such as coffee, tea, and wine, while their intake can be increased by various nutraceuticals and supplements. The richest sources of polyphenols are cocoa products, dark colored berries, some seeds (flaxseed) and nuts, spices and dried herbs, as well as some vegetables (olive and artichoke) [10]. Polyphenols are gaining increasing attention because of their promising effects on human health. Mechanisms underlying their health benefits include, among others, antioxidant and anti-inflammatory activities [11]. The antioxidant activity of polyphenols relies on several mechanisms, including the scavenging of free radicals and reactive oxygen species, as well as the activation of antioxidant enzymes [12]. Thus, the efficacy of polyphenols as antioxidants depends on their ability to eliminate oxygen species and suppress the expression/activation of prooxidant genes on one hand and promote the expression/activation of antioxidant genes on the other [13]. Anti-inflammatory activity of polyphenols includes various mechanisms, such as the regulation of cytokine levels, regulation of pro-inflammatory gene transcription, and promotion of anti-inflammatory gene expression [14,15].

This review paper aimed to evaluate the effects of polyphenol-rich juices and beverages in adults with metabolic syndrome or related cardiometabolic phenotypes, such as insulin resistance, obesity with risk factors, dyslipidemia, impaired glucose regulation, or type 2 diabetes. Only human intervention studies were considered, preferably randomized controlled, crossover, and parallel-group designs, but also prospective uncontrolled interventions that undoubtedly defined beverage exposures. Selected studies needed to report effect on at least one prespecified outcome related to oxidative stress, inflammation, and cardiometabolic risk. We aimed to provide nutritionally relevant findings and potential recommendations on how to enhance the health quality of everyday diet, rather than to test the isolated compounds in the form of supplements. Considering the heterogeneity of the literature data regarding the sources of polyphenols and their dosages, we focused on one food matrix. Beverages were chosen as food sources with the least complex composition, ideally, with the lowest influence of other components (nutrients) on polyphenols’ biological activity. Also, polyphenol-rich drinks are convenient vehicles to deliver polyphenols into the everyday diet without changing eating habits tremendously. Among beverages, we focused on fruit (vegetable) juices to exclude other polyphenol-rich drinks—such as tea, wine, and coffee—and thereby reduce heterogeneity across studies.

## 2. Methods

### 2.1. Literature Search Strategy

This narrative review is grounded in a structured and focused search of the biomedical literature addressing polyphenol-rich juices and beverages in adults with metabolic syndrome and related cardiometabolic conditions. An electronic search was performed in PubMed/MEDLINE and was complemented by manual screening of the reference lists of key primary studies and relevant reviews to identify additional eligible trials. The search covered the period from database inception to May 2025. It combined controlled vocabulary (e.g., MeSH/Emtree terms) and free-text keywords capturing four conceptual domains: polyphenolic compounds (such as polyphenols, flavonoids, anthocyanins and proanthocyanidins), beverage matrices (e.g., juice, beverage, drink, fruit juice, vegetable juice), metabolic and cardiometabolic conditions (e.g., metabolic syndrome, MetS, insulin resistance, type 2 diabetes, obesity, dyslipidaemia, cardiometabolic risk), and redox or inflammatory outcomes (e.g., oxidative stress, antioxidant, lipid peroxidation, C-reactive protein, interleukins, tumor necrosis factor, VCAM-1, ICAM-1). These terms were combined using Boolean operators (AND/OR), and available database filters for human studies and adult populations were applied where appropriate. A formal language restriction was imposed, and only articles published in English were considered eligible for inclusion.

### 2.2. Eligibility Criteria and Study Selection

Although the present work is explicitly conceived as a narrative rather than a systematic review, the identification and selection of studies followed a transparent, a priori framework in order to provide a comprehensive yet thematically focused synthesis of human intervention data. Studies were considered eligible if they fulfilled several criteria simultaneously. First, with respect to design, only human intervention trials were included, encompassing randomized controlled, crossover, and parallel-group designs, as well as non-randomized or uncontrolled intervention studies that prospectively evaluated a defined beverage. Second, in terms of population, the review was restricted to adults with metabolic syndrome or closely related cardiometabolic phenotypes, including overweight or obese individuals with additional risk factors, patients with dyslipidaemia, impaired glucose regulation or type 2 diabetes, and subjects characterized by elevated cardiovascular risk. Third, regarding the intervention, the focus was on orally consumed polyphenol-rich juices or beverages derived from fruits or vegetables, such as orange, pomegranate, cranberry, bilberry, blueberry, strawberry, açaí, citrus–aronia formulations, beet leaves and stalks, or mixed vegetable/legume juices; in all cases, the product and dose had to be clearly described. Fourth, eligible studies were required to include either a placebo or low-polyphenol control beverage or, in uncontrolled designs, to permit comparison with pre-intervention (baseline) values. Finally, to be retained, each trial had to report at least one outcome within the prespecified domains of interest: cardiometabolic risk factors, oxidative stress–related parameters, or inflammatory and endothelial biomarkers.

Studies were excluded if they were conducted exclusively in vitro or in animal models; if they employed observational designs (cross-sectional, case–control or cohort); if the intervention consisted of isolated polyphenol supplements, capsules or extracts not administered in the form of juices or beverages; if they were conducted solely in healthy volunteers without defined cardiometabolic risk, except where the primary focus was on elevated cardiovascular risk profiles; or if they were available only as conference abstracts, case reports or non–peer-reviewed material lacking sufficient methodological detail.

Study selection proceeded in two stages. In the first stage, titles and abstracts retrieved by the electronic searches were screened to exclude clearly irrelevant records and to identify potentially pertinent intervention trials. In the second stage, full-text articles for all remaining records were obtained and assessed in detail against the eligibility criteria described above. Any discrepancies or uncertainties regarding inclusion were resolved by discussion among the authors until consensus was reached. The final set of trials that met all criteria is summarized in Table 1 and forms the evidentiary foundation for the narrative synthesis presented in the subsequent sections.

### 2.3. Data Extraction and Narrative Synthesis

From each eligible study, data were systematically extracted on study design (parallel versus crossover; randomized versus non-randomized; presence and nature of placebo or control arms), sample size, participant characteristics (including age, sex and underlying condition), details of the intervention (type of juice or beverage, botanical source, polyphenol-rich matrix, daily dose and duration of intake), comparator characteristics, and the predefined outcome measures covering nutrition status (anthropometric, biochemical, clinical, dietary), cardiometabolic risk factors, oxidative stress markers and inflammatory or endothelial biomarkers.

Given the pronounced heterogeneity across trials in terms of populations, beverage composition and polyphenol content, intervention doses and durations, as well as the diversity of analytical methods and outcome panels, a formal quantitative meta-analysis was neither feasible nor appropriate. Instead, a structured narrative synthesis was undertaken. Studies were grouped according to their primary outcome domain, and their findings were integrated qualitatively. Within each domain, particular emphasis was placed on the direction and internal consistency of effects across trials, the magnitude and statistical significance of reported changes, and the potential modifying influence of baseline risk profile, beverage matrix and composition, and intervention duration. This approach was chosen to accommodate heterogeneity while allowing a coherent, clinically oriented appraisal of the potential role of polyphenol-rich juices and beverages in the management of metabolic syndrome and related cardiometabolic conditions.

## 3. Effects on Cardiometabolic Risk Factors

Metabolic syndrome is commonly defined as a cluster of cardiometabolic risk factors, including abdominal obesity (defined as elevated waist circumference), increased blood pressure, high blood sugar level, increased blood triglyceride level, and low HDL cholesterol [2].

Most of the included trials tested the impact of polyphenol-rich juices on various anthropometric, biochemical, or clinical factors associated with metabolic syndrome and/or related conditions.

### 3.1. Effects on Anthropometric Outcomes

As previously stated, dietary polyphenols have a role in the prevention of obesity and obesity related conditions, which mostly rely on their anti-inflammatory and/or antioxidant effects. These effects can lead to weight loss, increased energy expenditure, and fatty acid oxidation, decreased blood lipids, and enhanced insulin sensitivity [39].

Consumption of orange juice with either normal or high polyphenol content (providing 299 mg and 745 mg of polyphenols/day, respectively) for 12 weeks significantly reduced body weight, body mass index (BMI), and waist circumference (*p* < 0.001 for all three variables) in overweight/obese adult males and females (Table 1) [16].

Significant correlation of waist-to-hip ratio and metabolic syndrome has been reported in earlier studies. According to a cross-sectional study in Spanish adolescents, covering 18 secondary schools, the waist-to-hip ratio represents the superior predictor of MetS when compared to waist circumference and BMI [40]. In the study of Johnson et al. (2020), overweight/obese subjects consumed tart cherry juice (480 mL per day, providing more than one gram of total polyphenols) for 12 weeks (with tests at 6 and 12 weeks) [19]. Waist-to-hip ratio significantly decreased after 6 weeks (*p* = 0.003) in the intervention group, compared to the placebo group (Table 1) [19]. Compared to the above study with orange juice, tart cherry juice showed significance after a shorter period of duration, possibly due to the higher polyphenol dosage.

After 8 weeks of consuming orange juice being fortified with probiotics and vitamin D (250 mL per day), body weight (*p* = 0.001), BMI (*p* = 0.005), as well as hip circumference (*p* < 0.001) significantly decreased in overweight/obese subjects with hyperglycemia and/or hyperlipidemia, compared to a control group that consumed unfortified orange juice. In both groups, body fat mass significantly decreased (intervention group *p* = 0.003, control group *p* = 0.01) (Table 1) [17].

In abdominally obese men, following intake of low-calorie cranberry juice (providing approximately 460 mg of total polyphenols per day) for 4 weeks, body weight (*p* < 0.05), BMI (*p* < 0.05), and waist circumference (*p* < 0.0001) significantly decreased (Table 1) [20]. Similarly, body fat mass significantly decreased (*p* = 0.01) in obese subjects who consumed pomegranate juice (while it increased in the placebo group), and body weight and BMI showed trends toward a decrease (*p* = 0.089; *p* = 0.112, respectively) (Table 1) [28]. The latter two studies reported significance after a shorter period of intervention, compared to the above-listed interventions. This could potentially be attributed to the population enrolled, as these studies included obese subjects only, i.e., more homogeneous groups, that are at greater risk. Thus, significance is more likely to be observed than when enrolling in overweight subjects as well.

### 3.2. Effects on Biochemical Outcomes

Metabolic syndrome is closely associated with dyslipidemia, characterized by elevated plasma triglycerides and apolipoprotein B on one side, and lower HDL cholesterol on the other. Additionally, smaller and denser LDL particles typically appear in this condition, along with the corrupted anti-atherogenic activity of small HDL3, which aligns with lower HDL levels [41]. Regarding the impact on blood lipids, 8 weeks of low-calorie cranberry juice consumption (346 mg of polyphenols daily) significantly decreased the level of triglycerides (*p* = 0.027) in overweight/obese subjects, compared to the placebo group. Specifically, the participants with higher baseline concentrations were more likely to experience a larger treatment effect (Table 1) [21]. The same juice, after 4-week consumption, caused a trend in a decrease in triglyceride levels (*p* = 0.053) in abdominally obese men. In the same study, the concentration of HDL cholesterol significantly increased after 4-week consumption of both 250 mL (200 mg of polyphenols) and 500 mL (400 mg of polyphenols) daily doses (*p* < 0.01; *p* < 0.0001, respectively) [20].

Additionally, blood triglycerides, along with the apo B concentrations, were significantly reduced (*p* = 0.049 and *p* ≤ 0.001, respectively) after 12 weeks of orange juice (providing 299 mg of total polyphenols daily) intake in overweight/obese subjects [16]. These studies have indicated that the duration of intervention might not be detrimental regarding the effect on blood lipids. On the other hand, the dose of polyphenols applied in these studies was similar, ranging from 200 to 400 mg per day. Unexpectedly, 12 weeks of intervention with tart cherry juice, although providing polyphenols in higher doses (approximately a gram per day), only tended to decrease blood total cholesterol level (*p* = 0.08) [19]. This could be related to the differences in baseline values of tested parameters.

Unlike the inconsistent data for berry juices, the citrus juices seemed to be the more effective in regard to the impact on blood lipids. Additionally, two studies have reported the beneficial effects of citrus-based beverages on blood cholesterol. Specifically, eight-week-long consumption of orange juice fortified with vitamin D and probiotics significantly decreased concentrations of all three cholesterol parameters (total cholesterol, *p* = 0.05; LDL, *p* = 0.004; HDL, *p* = 0.01) [17]. Since this juice was fortified, the observed effects could rely on synergistic effects of added ingredients. Finally, the 6-month-long intake of citrus-based juice enriched with polyphenols from aronia significantly reduced total, as well as LDL and HDL cholesterol (*p* < 0.05 for all three) in subjects with metabolic syndrome (Table 1) [18]. This juice was enriched with aronia extract, which is potential hypolipidemic agent, and the observed effects could not be attributed to citrus juices solely but rather be the result of synergistic interactions.

**Figure 1:**
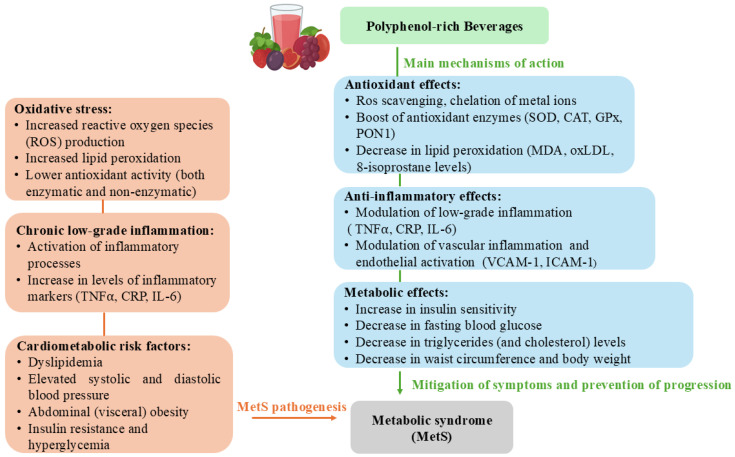
The main (and possible) mechanisms of polyphenol-rich beverages impact on metabolic syndrome (MetS) features and pathogenesis.

Insulin resistance is commonly defined as the inability of insulin to transport glucose inside the cell, which eventually causes hyperinsulinemia and impaired glucose tolerance. Besides this, insulin resistance in fat tissues leads to defective insulin-mediated inhibition of lipolysis. The increase in free fatty acids that consequently occurs alters the insulin signaling cascade in various organs and, thus, worsens insulin resistance in turn. Besides an increase in blood lipids, insulin resistance contributes to metabolic syndrome via its impact on hypertension development [42]. Some of the included studies showed beneficial effects of polyphenol-rich beverages on insulin resistance and blood sugar levels (Figure 1). Plasma insulin concentration decreased significantly (*p* = 0.04) after 12-week consumption of orange juice with normal polyphenol concentration (299 mg daily) in overweight/obese subjects. At the same time, it tended to decline (*p* = 0.06) after intake of juice with a high concentration (745 mg) of polyphenols [16]. Plasma insulin and homeostasis model assessment of insulin resistance (HOMA-IR) significantly dropped (*p* = 0.04; *p* = 0.02, respectively) in a study that tested the 8-week impact of orange juice fortified with probiotics and vitamin D, in overweight/obese subjects with hyperglycemia and/or hyperlipidemia [17]. These studies tested the impact of orange juice, rather than isolated orange polyphenols. In the first study, both insulin and glucose concentrations were within normal ranges at the beginning and the end of intervention, while the HOMA-IR index, as a measure of insulin sensitivity, remained unchanged [16]. Bearing in mind the antidiabetic properties of isolated citrus polyphenols—hesperidin and naringenin, it should be established whether the food matrix could modulate these effects [43]. Orange juice, like any other fruit juice, represents a complex matrix, containing carbohydrates, including fructose, as the major macronutrients. It is well established that dietary fructose promotes insulin resistance via several metabolic pathways that interplay and lead to increased blood glucose and insulin levels, and decreased insulin sensitivity [44]. Thus, we could speculate that the food matrix, fructose specifically, could weaken the effect of polyphenols from orange and other fruit juices on insulin sensitivity.

Acute reducing effects on insulin level (*p* = 0.013) and insulin resistance (*p* = 0.003) were reported after intake of *Passiflora setacea* pulp in overweight or slightly obese men (Table 1) [35]. Homeostasis Model Assessment of Beta-cell function (HOMA-%B) was significantly increased after 6 (*p* = 0.002) and 12 weeks (*p* = 0.035) of tart cherry juice consumption in subjects with MetS [19]. Significant acute effects on blood sugar level were reported in two studies, including type 2 diabetes subjects, testing the impact of juice made from spinach, broccoli, celery, green beans, and chickpea (4 weeks), and pomegranate juice (8 weeks), respectively (Table 1) [29,36]. Low-calorie cranberry juice consumption significantly decreased fasting plasma glucose, along with the HOMA-IR, after 8 weeks of regular consumption in overweight/obese subjects [21].

### 3.3. Effects on Blood Pressure

Metabolic syndrome is closely associated with increased blood pressure. A recent study has demonstrated a significant correlation between MetS and average 24 h SBP, office and home-monitored SBP, as well as daytime and nighttime ambulatory SBP [45]. Additionally, an earlier study reported a two times higher risk of new onset of home, office, or ambulatory hypertension in subjects with MetS, compared to those without [46].

Included studies showed inconsistent data regarding the impact of polyphenol-rich beverages on blood pressure in MetS and related conditions. Based on the observations, it is not possible to ascertain whether the dosage, type, and/or duration of intervention are of decisive importance for the effect on blood pressure. In a study with overweight/obese subjects, orange juice consumption for 12 weeks (providing 299 mg of polyphenols daily) significantly decreased both SBP and diastolic blood pressure (SBP, *p* = 0.009; DBP, *p* ≤ 0.001) [16]. The same effect (*p* = 0.001 for both SBP and DBP) was reported in a placebo-controlled study that tested the impact of natural pomegranate juice intake for one week in subjects with MetS (Table 1) [31]. Significant drop in DBP was reported after an acute intake of both low-dose and high-dose (32 and 77.5 mg of total phenolics, respectively) of organic beet leaves and stalks juice in adults with dyslipidemia (Table 1) [37]. On the contrary, SBP showed a non-significant (*p* = 0.07) decrease at 8 weeks of intervention with low-calorie cranberry juice (providing 458 mg of total polyphenols daily) [22]. Pomegranate juice intervention showed the same trend after 6 weeks of consumption [30]. Both studies included females with MetS (Table 1).

Although the primary interventions in MetS subjects include lifestyle change, more precisely improved health quality of foods, calorie restriction, and enhanced physical activity, drug treatment is often recommended. First-line treatment for high cholesterol includes statin therapy, while triglyceride-lowering drugs include niacin and fibrates, and metformin is commonly applied for glucose lowering. Regarding the treatment of hypertension, besides calorie restriction, investigators recommend ACE inhibitors, angiotensin receptor blockers, and calcium channel blockers as the first-line treatment, as they do not provoke insulin resistance [47].

A recent review has discussed the potential interactions of statins and polyphenol-based supplementation. Interactions were found in more than 80% of the studies included. Polyphenols significantly affected statin pharmacokinetics, altering total drug exposure, either by enhancement or inhibition, but only 5 studies addressed interactions at the clinical level, leading to conflicting findings on the potential impact on therapeutic efficacy and adverse effects [48]. Polyphenols interact with metformin in several ways, depending on the type and food source. For instance, one study suggested a potential synergistic role of polyphenols from a blend containing berries like aronia and black currant in enhancing the efficacy of metformin [49]. On the other hand, intake of large amounts of polyphenol-rich beverages with metformin could potentially lead to the risk of hypoglycemia. Regarding the interactions with antihypertensive drugs, specifically angiotensin-converting enzyme (ACE) inhibitors, polyphenols have similar mechanisms of action to these drugs. Specifically, inhibition of ACE is one of the features determining polyphenols’ blood pressure-lowering effects, and, thus, they could act synergistically with these drugs [50].

It is important to note that out of the included studies, most listed medications as exclusion criteria (13 out of 23), while other studies have not mentioned the use of drugs (Table 1). Only one study reported the use of blood pressure-lowering drugs, specifically ACE inhibitors [29]. Few studies included subjects with type 2 diabetes, and underlined use of insulin as exclusion criteria, implying oral antidiabetic medications were allowed, although this was not clearly reported in all studies (Table 1).

## 4. Effects on Oxidative Stress

While citrus juices showed the most promising results in case of cardiometabolic risk factors (including anthropometric indices, blood lipids, and insulin), pomegranate juice and berries showed stronger antioxidant, as well as anti-inflammatory potential. Still, it is hard to compare the effects of different polyphenol-rich beverages on oxidative stress, as not all of them tested the same indicators. Beside the heterogeneity in polyphenols dosage and intervention duration, study populations differed in terms of oxidative baseline status. Mostly, the effects were, as expected, more pronounced in subjects with greater oxidative damage at the baseline.

Bioavailability of polyphenols is another factor that could contribute to the varying effects among different polyphenol-rich beverages. As bioavailability varies greatly among different polyphenols, those in higher dosages are not necessarily those leading to the highest levels of active metabolites in the blood and target cells, i.e., are not necessarily the most effective ones. While gallic acid (present in fruits, including berries) and isoflavones are identified as the most well-absorbed polyphenols, followed by flavanones (citrus juices being one of the main sources), catechins, and quercetin glucosides, the least well-absorbed polyphenols are the proanthocyanidins and anthocyanins (present in high amounts in berries and pomegranate) [51].

### 4.1. Effects on Blood Antioxidants and Total Antioxidant Activity

In MetS and related conditions, a balance between antioxidants and (pro)oxidants in circulation and tissues can become disturbed, leading to increased oxidative damage of biomolecules [52].

In the included studies, inconsistent data were reported regarding the potential impact of polyphenol-rich beverages on total antioxidant capacity (TAC), reflecting the level of all individual antioxidants in plasma. Additionally, the effect was not dependent on the dose of polyphenols nor on the duration of the intervention. It appeared that berries and pomegranate juice were the most effective in the case of TAC improvement. Specifically, in females with MetS consuming low-calorie cranberry juice for 8 weeks (daily dose of total phenolics 458 mg), plasma antioxidant capacity improved by 47%. This was a significant (*p* < 0.05) effect, especially when compared to the placebo group, in which only a 7% increase was detected [22]. Cranberry juice changed plasma antioxidant capacity in men with abdominal obesity, as well. The participants successively consumed 125 mL, 250 mL, and 500 mL of juice for 4 weeks each. After a nearly significant increase (*p* = 0.0619) with the lowest dose (100 mg of total polyphenols per day), antioxidant capacity decreased significantly at both higher doses (*p* < 0.05 for both) [20]. Compared to the placebo group, total antioxidant capacity significantly increased (*p* < 0.05) after 6 weeks of pomegranate juice consumption (425 mg of total polyphenols/day) in participants with type 2 diabetes (Table 1) [32]. On the contrary, this parameter remained unchanged in men and women with MetS after regular consumption of tart cherry juice (containing 1070 mg of polyphenols in a daily dose) for 12 weeks [19].

Another method for the estimation of antioxidant power is the measurement of the ferric reducing ability of plasma, by applying the FRAP assay. Two included studies analyzed the effects of polyphenol-rich beverages on FRAP in plasma. While 4 weeks of pomegranate juice (containing 2600 ppm gallic acid equivalents) consumption increased FRAP significantly (by 35%) in diabetic subjects [33], 4 weeks of bilberry juice intake in subjects at elevated risk of cardiovascular disease caused no change in FRAP (Table 1) [23]. It remains uncertain what led to these opposite findings. Both studies tested a relatively short period of intervention, but no data on the amount of total polyphenols in the second study are given. In the first study, a significant increase in total thiols’ level was observed (by 25%) as well. Both FRAP and thiols’ levels remained increased after a 4-week wash-out period (Table 1) [33].

Regarding the effects on non-enzymatic blood antioxidants, scarce data are provided. Specifically, the polyphenol-rich jelly drink significantly increased glutathione levels in adults with dyslipidemia after 8 weeks of intake [38]. Twelve weeks of orange juice consumption (providing 745 mg of polyphenols per day) significantly improved coenzyme Q10 status in obese/overweight subjects. In contrast, a lower dose of polyphenols (299 mg per day) significantly increased β-carotene level [39].

### 4.2. Effects on Antioxidant Enzymes

Altered activities of antioxidant enzymes in MetS subjects were previously investigated. Patients with MetS were found to have higher activities of superoxide dismutase and glutathione reductase on one side, and lower activities of catalase and paraoxonase-1 on the other when compared to age and sex matched controls [6]. Not many studies have analyzed the effects of polyphenol-rich juices and beverages on antioxidant enzymes [53]. While berries and pomegranate seemed to be the most potent in the case of boosting nonenzymatic defense, orange juice had an effect on antioxidant enzymes. Pomegranate juice, on the other hand, significantly affected Paraoxonase-1 (PON1) activity as well.

Specifically, orange juice with both normal and high doses of polyphenols (299 or 745 mg per day) caused a significant decrease in the activities of erythrocyte glutathione reductase and catalase after 12 weeks of consumption in overweight/obese subjects (Figure 1). This is not in accordance with our previous research in which glucomannan-enriched aronia juice significantly increased glutathione peroxidase in abdominally obese women, while activities of catalase and superoxide dismutase remained unchanged [49]. Limited literature data and somewhat ambiguous findings prevent us from making any conclusions regarding the potential beneficial impact of polyphenol-rich drinks on erythrocyte antioxidant enzymes.

As reported in two studies, consumption of pomegranate juice resulted in a significant increase in PON1 activity in subjects with type 2 diabetes [32,33]. Paraoxonase-1, an antioxidant enzyme synthesized in the liver, primarily neutralizes lipid peroxides and prevents LDL oxidation, and, thus, ameliorates oxidative damage of cells [54]. It has been reported that pomegranate polyphenols beneficially affect PON-1 activity by increasing the expression of its mRNA and protein levels in the liver (Figure 1) [55].

### 4.3. Effects on Parameters of Lipid Peroxidation

Oxidative damage to lipids is typically initiated by free radicals, resulting in alterations to membrane lipids and accumulation of toxic compounds. Hydroxyl radicals and hydrogen peroxide are considered key initiators of the lipid peroxidation process. To terminate the chain reaction of lipid peroxidation, two radicals can neutralize each other, or an antioxidant can neutralize a radical [56]. Polyphenols can act directly by scavenging radicals or indirectly by chelating metals involved in the initial phase of radical production (Figure 1) [12].

Markers of lipid oxidative damage commonly analyzed in interventional studies involving antioxidant supplementation include levels of malondialdehyde (MDA), hydroxynonenal (HNE), oxidized low-density lipoprotein (ox-LDL), and 8-isoprostane in urine (Figure 1). The consumption of 300 mL of pomegranate juice for 6 weeks resulted in a decrease in TBARS adducts of MDA (*p* < 0.05) in erythrocytes, with no similar changes found in plasma for TBARS-MDA of women with MetS [30]. Basu et al. (2011) have explored the effects of cranberry juice on plasma MDA and HNE levels [22]. The mean values for plasma MDA and HNE were reduced by about 50% after 8 weeks of treatment with 240 mL of the juice in women with MetS [22]. In a study examining the effects of orange juice treatment, a positive association was observed between hesperidin levels and MDA in plasma; however, MDA levels were not significantly decreased compared to values before and after 12-week treatment with either orange juice with high polyphenol or normal polyphenol content [16]. Khongrum et al. (2022) conducted a trial where the participants were randomly assigned to consume either the roselle calyces extract-enriched passion fruit jelly or a placebo drink for 8 weeks [38]. The treatment induced a significant alteration of MDA levels (*p* < 0.001) [38]. In diabetic patients, the levels of TBARS reactive substances have decreased compared to baseline values after 2 (by 19%) and 4 weeks (by 35%) of treatment with pomegranate juice [33]. The same authors have also observed a favorable effect on AAPH-induced lipid peroxidation after 4 weeks of treatment.

While pomegranate juice was the most effective one in terms of the impact on MDA, berry juices stood out in terms of their effects on MDA combined with other peroxidation biomarkers. One research group has examined the impact of juice consumption on combined markers of MDA and HNE, demonstrating favorable changes following treatment with cranberry [22], blueberry [24], and strawberry freeze-dried powder in patients with MetS (Table 1) [25]. The consumption of blueberry juice and strawberry powder resulted in a reduction of 0.2 μmol/L (*p* < 0.01) in the combined MDA and HNE marker, while cranberry juice led to a decrease of 1.7 μmol/L (*p* < 0.05) in men and women with MetS. The duration of the interventions varied across studies, with 4 weeks for strawberry powder and 8 weeks for cranberry and blueberry juices. Notably, the concentration of polyphenols was higher in the blueberry-treatment study (1624 mg) compared to the other two (229 mg each), suggesting that the response may not be directly related to the dosage of polyphenols (Table 1). However, baseline levels of MDA and HNE were significantly higher in the cranberry juice study, indicating that patients with greater lipid damage could potentially benefit more from supplementation with polyphenol-rich berry juices.

The levels of ox-LDL have been shown to change in several studies. For example, in an 8-week study, a significant reduction in plasma ox-LDL was observed in the blueberry beverage group, with a decrease of approximately 30 U/L compared to a reduction of 9.6 U/L in the control group of adults with MetS (Table 1) [24]. Furthermore, the consumption of citrus-based juice enriched with *Aronia melanocarpa* extract over six months led to decreased plasma ox-LDL levels in patients with MetS whose baseline ox-LDL levels were higher compared to healthy controls [18]. Conversely, treatment of overweight and obese patients with orange juices containing two different concentrations of polyphenols did not result in significant modifications of plasma ox-LDL levels [16].

The changes in urinary levels of 8-isoprostane, one of the most reliable biomarkers of lipid peroxidation due to its stability and specificity, have been examined in several intervention studies involving polyphenol-rich juices. Intervention with both regular and high-polyphenol orange juices led to a reduction in 8-isoprostane (prostaglandin F2α) levels in non-smoking obese/overweight adults after 12 weeks of treatment [16]. Additionally, treatment of male and female patients with MetS with acai juice (providing ~740 mg/day of polyphenols) for 12 weeks significantly altered the levels of their oxidative stress biomarker, urinary 8-isoprostane (Table 1) [26].

Previous studies conducted by our research group have demonstrated that polyunsaturated fatty acid (PUFA) profiles are associated with the levels of lipid peroxidation measured in serum/plasma and erythrocytes [57]. Moreover, treatment of different subject groups with aronia juice, rich in polyphenols, has led to favorable changes in both redox status and PUFA content. In studies related to metabolic disorders, fatty acid (FA) profiles have been analyzed, such as in Kojadinovic et al.’s research (2016), which reported beneficial alterations in PUFA profiles following treatment with pomegranate juice in patients with MetS [30].

The studies included in this review cover a wide range of polyphenol dosages, as well as the duration of intervention. Moreover, the effective dosage differed among the investigated parameters. In case of anthropometric parameters (body weight, BMI, and waist circumference), orange juice was effective in both low and high dosages of polyphenols (299 mg, 745 mg, respectively), cranberry juice in a dosage of 460 mg, while tart cherry juice in a dosage of 1 g was efficient in decreasing waist/hip ratio. The dosage of 200 to 400 mg was effective in decreasing TG levels and increasing HDL cholesterol, while tart cherry juice in a dosage of more than 1 g was not effective, suggesting that there might be some saturation effect. Regarding the blood insulin, a lower dose of polyphenols (299 mg) in orange juice seemed to be more effective than the higher dose. The effects on blood pressure were quite inconsistent, while lower dosages and intervention duration seemed to be more effective. It could be suggested that the effect depended more on the baseline levels of tested parameters, specifically blood pressure levels, than on the phenolic dosage.

Regarding the impact on oxidative stress, different parameters were differently influenced. Total antioxidant capacity was increased in two studies with similar dosage (approx. 400–450 mg), while in one study higher dosage had no effect (more than 1 g). As for the antioxidant enzymes, both doses of polyphenols (299 or 745 mg per day) from orange juice caused a significant decrease in glutathione reductase and catalase activities, while only the higher dosage increased superoxide dismutase activity.

Other included studies showed no significant effect on these parameters, although they have included similar or even higher dosages of polyphenols. The effective dosage might depend on the baseline characteristics of the participants (whether they are characterized by increased cardiometabolic risk, high inflammation, and oxidative stress), as well as the food (beverage) matrix.

Regarding food matrix and interactions with other nutrients, synergistic effects, as opposed to the previously mentioned antagonistic interactions, could be expected as well. For example, orange (citrus) juice, besides flavanones, contains vitamin C (ascorbic acid) and folate in considerable amounts. All these compounds are exerting promising anti-inflammatory and antioxidant activities [58]. Additionally, berry juices, as well as pomegranate juice, are good sources of vitamin C. Further on, the antihypertensive effects of polyphenol-rich juices could be enhanced by other ingredients, like mineral potassium. Specifically, aronia berry juice was reported to contain significant amounts of this mineral, which could contribute to the hypotensive effects of the present polyphenols [59].

## 5. Effects on Inflammation

Chronic low-grade inflammation is an essential hallmark of MetS, acting in concert with oxidative stress to exacerbate cardiometabolic risk. Within this context, polyphenol-rich juices and beverages have been increasingly investigated for their immunomodulatory and endothelial-stabilizing properties (Figure 1). The available body of evidence, though heterogeneous, converges on the notion that these bioactive-rich interventions can attenuate systemic inflammatory activity, particularly as reflected in circulating cytokines, adhesion molecules, and acute-phase reactants [60].

C-reactive protein (CRP), the archetypal biomarker of systemic inflammation, emerges as one of the most responsive indicators in intervention studies. Significant reductions were documented following medium- to long-term intake of polyphenol-rich juices: citrus-based formulations fortified with aronia extract lowered CRP alongside homocysteine after six months in patients with MetS [18], while pomegranate juice elicited consistent decreases in CRP both in MetS and type 2 diabetes populations (Table 1) [31,34]. Bilberry juice further extended these findings, producing reductions in CRP in subjects with elevated cardiovascular risk [23]. Conversely, no significant changes were detected in trials employing blueberry- or strawberry-based beverages [24,25], suggesting that the efficacy of polyphenolic interventions is not universal but depends critically on phytochemical composition and matrix effects.

Beyond CRP, several trials interrogated the cytokine milieu with compelling though varied results. Pomegranate juice was shown to lower circulating IL-6 and TNF-α in individuals with type 2 diabetes [57], while a polyphenol-rich jelly drink reduced TNF-α after eight weeks in dyslipidemic adults [38]. An acai beverage (delivering ~740 mg/day of polyphenols) significantly decreased interferon gamma (IFN-γ), a central amplifier of pro-inflammatory signaling [26]. Smaller or marginal effects were reported elsewhere: tart cherry juice induced only a borderline reduction in TNF-α in overweight participants with chronic low-grade inflammation (Table 1) [27]. Intriguingly, bilberry juice lowered IL-6, IL-15, and MIG concentrations, but paradoxically increased TNF-α, underscoring the complexity and possible bidirectional nature of immunological modulation by distinct phytochemical profiles [23].

Markers of vascular inflammation and endothelial activation also appeared sensitive to polyphenolic intervention (Figure 1). Both tart cherry juice [19] and cranberry juice [21] significantly reduced vascular cell adhesion molecule 1 (VCAM-1), a key mediator of leukocyte adhesion and vascular dysfunction, while ICAM-1 remained unaffected in the latter trial, suggesting selective sensitivity of adhesion molecules to polyphenolic interventions. These findings hint at a potential vascular-protective role mediated through attenuation of endothelial activation pathways, with implications for reducing atherosclerotic risk.

The collective evidence across the included studies portrays polyphenol-rich juices and beverages as consistent, though not uniformly potent, modulators of low-grade inflammation in cardiometabolic contexts. Rather than suggesting universal efficacy, the trials converge on a pattern of biomarker attenuation that is most evident for systemic reactants and endothelial activation markers. At the same time, effects on broader cytokine networks remain more variable. The aggregate pattern suggests several practical inferences. First, effect sizes tend to be modest to moderate but are most apparent in participants with higher baseline inflammation or cardiometabolic risk, indicating a potential “responder” phenotype. Second, matrix and composition matter: preparations standardized for higher polyphenol content or combined with co-actives more often yielded statistically robust changes in CRP, cytokines, or adhesion molecules than lower-polyphenol comparators or fruit types with weaker evidence. Third, duration and dose appear consequential: multiweek exposures generally outperform acute challenges, and studies employing higher daily polyphenol loads report more consistent biomarker shifts.

## 6. Conclusions

Metabolic syndrome is a complex pathophysiologic state characterized by insulin resistance, abdominal obesity, hyperlipidemia, and hypertension. Although there are different definitions of this condition derived by different health organizations, the variations are minor, and all underline the presence of cardiometabolic risk factors. Besides this, both oxidative stress and chronic low-grade inflammation represent essential hallmarks of MetS, which act in accordance with aggravating cardiovascular risk. As in any other pathological condition, lifestyle changes are recommended as the first-line interventions for management and eventual reversal of MetS. This includes changes in diet, with increased consumption of functional foods and ingredients, such as polyphenols- secondary plant metabolites with promising antioxidant and anti-inflammatory activities. In this review paper, we have summarized available data from human intervention studies regarding the potential health benefits of polyphenol-rich juices and beverages on subjects with MetS and related conditions.

Taken together, the obtained data support the adjunctive use of polyphenol-rich juices within comprehensive lifestyle management of MetS to attenuate oxidative stress and low-grade inflammation and improve present cardiometabolic risk factors. However, heterogeneity across the studies, including variability across products, doses, and populations, argues for caution in overgeneralization. Other limitations include the lack of standardized polyphenol quantification, as well as the fact that some studies did not report the polyphenol content at all; the relatively small sample sizes in several trials; and the absence of a placebo control (or the use of water as the control beverage). Additionally, some studies either allowed the use of medications for cardiometabolic comorbidities or did not clearly state whether such medications were excluded. Finally, several trials evaluated fortified products or combinations of different polyphenol-rich sources, which may further limit comparability across studies.

Future trials should adopt standardized polyphenol quantification, predetermine core inflammatory panels and parameters of oxidative stress, stratify by baseline inflammatory/oxidative status and medication use, and extend follow-up to test durability and clinical salience. As we have observed differences in efficient dosages, further research should aspire to identify the minimal effective dose and intervention duration, preferably by focusing on one group or one specific parameter (metabolic syndrome feature). Potential interactions with drugs, if subjects are prescribed them, should also be considered. Food matrix (composition) is another important determinant that should be addressed in future research. Designing a placebo that should mimic all compounds and features of tested beverages, but excluding polyphenols, should be adopted as a standardized goal. Future trials should strive for homogeneity in terms of beverage composition, polyphenols dosage, populations enrolled, and methods applied, which could potentially provide a foundation for formal quantitative meta-analysis. Until such data is available, clinicians and researchers may reasonably view high-polyphenol juices as evidence-supported, low-risk dietary complements with the greatest likelihood of benefit in individuals manifesting elevated inflammatory and oxidative stress markers at baseline.

## Figures and Tables

**Table 1:** Summary of interventional trials with polyphenol-rich juices on cardiometabolic risk factors.

Ref.	Type of Study	Number of Patients	Patients’ Characteristics	Use of Medications	Age (Years)	Intervention	Dose	Duration	Results
**Citrus-based beverages:**									
[16]	Randomized parallel	100	Overweight or obese adults	NR (*Not reported*)	18–65	Orange juice with a normal or high concentration of polyphenols	2 × 250 mL/day	12 weeks	↓ Weight, BMI, WC (both); ↑ Glucose (both); ↓ Insulin (NPJ, trend HPJ); ↑ HOMA-IR (HPJ); ↓ TG, ↓ apoB (NPJ); ↑ apoA-I (HPJ); ↓ Leptin (both); ↓ SBP, DBP (NPJ); ↓ GR, ↓ CAT (both); ↑ SOD (HPJ); ↑ CoQ10 (HPJ); ↑ β-carotene (NPJ); diff. in CoQ9; ↓ 8-OHdG, ↓ 8-iso-PGF_2_α (both); ↓ Urinary 8-OHdG (HPJ < NPJ); ↓ Plasma LPO (NPJ); LPO diff. between interventions
[17]	Randomized parallel	50	Increased BMI, hyperglycemia and/or hyperlipidemia and/or hypertension	NO	18–70	Fortified orange juice or normal orange juice	250 mL/day	8 weeks	↓ Weight, BMI, HC, insulin, HOMA-IR, total-C, LDL-C, HDL-C (enriched OJ); ↑ FGI, ↑ QUICKI; ↓ SBP (both); ↓ DBP (enriched OJ only); ↓ PWV (control).
[18]	Placebo controlled	53	MetS	NR	50–65	Citrus-based juice (95% citrus juice + 5% Aronia melanocarpa extract) or a placebo	300 mL/day	6 months	↓ Total-C, LDL-C, HDL-C, ox-LDL, CRP, homocysteine (MetS group, after 4–6 mo CJ vs. baseline)
**Berries-based beverages:**									
[19]	Placebo controlled	29	MetS	NR	20–60	Tart cherry juice or placebo	480 mL/day	12 weeks	↓ Waist/hip ratio, ox-LDL, VCAM-1; ↑ HOMA-%B.
[20]	Non-randomized, uncontrolled	30	Sedentary, abdominally obese men, normolipidemic to mildly hyperlipidemic	NO	51 ± 10	Low-calorie cranberry juice cocktail	Escalated dose: 125 mL/day, 250 mL/day, 500 mL/day	12 weeks (3 × 4 weeks)	↓ Weight, BMI, WC; ↑ HDL-C; ↓ NOx; ↑ Antioxidant capacity (dose-dependent)
[21]	Placebo controlled	56	BMI between 20 and 38 kg/m^2^,	NO	25–65	Low-calorie cranberry juice or a placebo	2 × 240 mL/day	8 weeks	↓ TG, ↓ FPG, ↓ HOMA-IR, ↓ CRP, ↓ DBP; ↑ insulin sensitivity (LCCJ vs. placebo)
[22]	Placebo controlled	29	MetS	NO	20–60	Low-calorie cranberry juice or a placebo	480 mL/day	8 weeks	↑ plasma antioxidant capacity (spectrophotometric), ↓ ox-LDL (ELISA), ↓ MDA (spectrophotometric) (vs. placebo)
[23]	Randomized parallel	62	Patients with an elevated risk of CVD	NO	30–68	Bilberry juice	330 mL/day	4 weeks	↓ CRP, ↓ IL-6, ↓ IL-15, ↓ MIG, ↑ TNF-α, ↑ plasma quercetin and p-coumaric acid (vs. baseline)
[24]	Randomized parallel	66	MetS	NO	45–55	Freeze-dried blueberries reconstituted with water	50 gr + 960 mL/day	8 weeks	↓ SBP, ↓ DBP, ↓ ox-LDL, ↓ combined MDA + HNE (greater reduction vs. control)
[25]	Uncontrolled study	16	Mets women	NO	51 ± 9.1	Freeze strawberries powder + water + Splenda + vanilla essence	2 cups/day	4 weeks	↓ total-C, ↓ LDL-C, ↓ MDA, ↓ HNE (vs. baseline)
[26]	Placebo controlled	37	MetS	NR	18–65	Acai beverage or placebo	650 mL/day	12 weeks	↓ IFN-γ, ↓ 8-isoprostane (vs. placebo)
[27]	Placebo controlled, cross-over	10	Overweight and obese adults	NO	38.1 ± 12.5	Tart cherry juice or placebo	240 mL/day	4 weeks	↓ MCP-1, ↓ erythrocyte sedimentation rate, ↓ TNF-α (vs. placebo)
**Pomegranate-based beverages:**									
[28]	Placebo controlled	20	Obese adults	NO	25–55	Commercial pomegranate juice or placebo	120 mL/day	1 month	↓ Weight, ↓ adiposity (PJ vs. baseline, NS vs. placebo); ↔ other metabolic markers
[29]	Randomized parallel	85	Patients with type 2 diabetes	YES (ACE inhibitors)	37–60	Fresh pomegranate juice	1.5 mL/kg body weight, single dose	Single day	↓ fasting glucose, ↓ insulin, ↓ HOMA-IR, ↑ HOMA-%β (3 h post–single-dose juice vs. baseline, T2D patients)
[30]	Randomized parallel	23	MetS women	NO	40–60	Pomegranate juice	300 mL/day	6 weeks	↓ SBP (trend, non-significant; vs. baseline)
[31]	Placebo controlled	30	MetS	NO	51.57 ± 10.04	Natural pomegranate juice	500 mL/day	7 days	↓ SBP, ↓ DBP, ↓ hs-CRP; ↑ TG, ↑ VLDL-C (vs. placebo)
[32]	Randomized controlled	60	Patients with type 2 diabetes mellitus	NR (Insulin excluded)	54.6 ± 8.4	Pomegranate juice	200 mL/day	6 weeks	↑ total antioxidant capacity, ↑ PON1 activity, ↓ ox-LDL, ↓ anti-oxLDL antibodies (vs. baseline, PJ group)
[33]	Parallel	30	Patients with type 2 diabetes mellitus	NR	50–60	wonderful variety pomegranate juice, or wonderful variety pomegranate juice liquid extract	50 mL/day; 5 mL/day	4 weeks; 6 weeks	↑ PON1 (aryltransferase, paraoxonase, lactonase), ↓ TBARS (−19% at 2 wk; −35% at 4 wk; −26% post-washout), ↓ AAPH-induced lipid peroxidation, ↑ total thiols (+25%), ↑ FRAP (+35%; +59% post-washout) (vs. baseline)
[34]	Placebo controlled	44	Patients with type 2 diabetes mellitus	Oral hypoglycemic (Insulin excluded)	40–65	Commercial pomegranate juice	250 mL/day	12 weeks	↓ hs-CRP, ↓ IL-6, ↓ TNF-α (vs. placebo)
**Other polyphenol-rich beverages:**									
[35]	Placebo controlled	23	Overweight or slightly obese men	NR	40–64	*Passiflora setacea* pulp, or placebo	150 gr or 50 gr	2 weeks	↓ glucose, insulin, HOMA-IR; ↑ IL-2 (acute phase); ↑ glucose, HOMA-IR, IL-6 (chronic phase) (vs. placebo)
[36]	Uncontrolled parallel	36	Patients with type 2 diabetes (6–10 years)	NR	40–50	Juice prepared from (300 gr spinach, 200 gr broccoli, 50 gr celery, 200 gr green beans, 50 gr chickpea)	300 mL/day	4 weeks	↓ postprandial glucose spike at 60 min and 120 min (vs. baseline, juice group)
[37]	Placebo controlled	13	Adults with diagnosed dyslipidemia	NO	20–59	Organic beet leaves and stalks juice (BLS); derived from Beta vulgaris or a placebo	Low/high BLS, dose NR (*NR = not reported*)	Single day	↑ glucose and insulin at 30 min (all groups); ↓ DBP (within both BLS groups vs. baseline); attenuation of HDL-C drop post–high-fat meal (vs. placebo)
[38]	Placebo controlled	40	Patients with dyslipidemia	NO	35–60	Jelly drink containing polyphenol-rich roselle calyces extract and passion fruit juice with pulp concentrate or placebo	300 mL/day	8 weeks	↓ LDL-C, ↓ TG, ↓ TNF-α, ↓ MDA, ↑ GSH (vs. placebo)

**Legend:** Abbreviations—apoA-I: apolipoprotein A-I; apoB: apolipoprotein B; AAPH: 2,2′-azobis(2-amidinopropane) dihydrochloride; BMI: body mass index; BLS: beet leaves and stalks; CAT: catalase; CJ: citrus juice; CoQ9/10: coenzyme Q9/10; CRP: C-reactive protein; DBP: diastolic blood pressure; FGI: fasting glucose index; FRAP: ferric reducing antioxidant power; GSH: glutathione; GR: glutathione reductase; HDL-C: high-density lipoprotein cholesterol; HC: hip circumference; HNE: 4-hydroxynonenal; HOMA-IR: homeostatic model assessment of insulin resistance; HOMA-%β: homeostatic model assessment of β-cell function; HPJ: high-polyphenol orange juice; IFN-γ: interferon-gamma; IL: interleukin; LDL-C: low-density lipoprotein cholesterol; LCCJ: low-calorie cranberry juice; LPO: lipid peroxidation; MDA: malondialdehyde; MCP-1: monocyte chemoattractant protein-1; MIG: monokine induced by interferon-gamma; MetS: metabolic syndrome; NOx: nitric oxide metabolites; NPJ: normal-polyphenol orange juice; ox-LDL: oxidized low-density lipoprotein; PJ: pomegranate juice; PON1: paraoxonase-1; PWV: pulse wave velocity; QUICKI: quantitative insulin sensitivity check index; SBP: systolic blood pressure; SOD: superoxide dismutase; TBARS: thiobarbituric acid reactive substanc; T2D: type 2 diabetes; TG: triglycerides; TNF-α: tumor necrosis factor-alpha; VCAM-1: vascular cell adhesion molecule-1; WC: waist circumference; Total-C: total cholesterol. **Notes:** Arrows indicate direction of change (↑ increase, ↓ decrease, ↔ no change). “vs. baseline” denotes comparison to pre-intervention values; “vs. placebo/control” denotes between-group differences.

## Data Availability

No new data were created or analyzed in this study. Data sharing is not applicable to this article.
